# Early Chest Imaging in Patients with Puumala Hantavirus Infection

**DOI:** 10.5334/jbsr.2039

**Published:** 2020-09-04

**Authors:** Olivier Lebecque, Ana Falticeanu, Cécile Abraham, Michael Dupont

**Affiliations:** 1 Université catholique de Louvain, CHU UCL Namur, Department of Radiology, 1 Avenue Dr G Thérasse, 5530, Yvoir, BE

**Keywords:** Puumala virus, hantavirus, thorax, hemorrhagic fever with renal syndrome, nephropathia epidemica

## Abstract

**Purpose:**

To describe early chest imaging abnormalities in patients with acute Puumala virus infection.

**Materials and Methods:**

This retrospective study (2005–2017) comprised 64 patients who were admitted to the emergency department of a Belgian hospital. These patients were diagnosed with serologically confirmed acute Puumala virus infection and had at least one chest X-ray (CRX). Imaging studies were evaluated by two experienced chest radiologists reaching agreement by consensus, and abnormalities were reported according to the Fleischner Society glossary of terms for thoracic imaging. When a patient underwent multiple CRX, only the findings of the first were recorded. Six patients underwent chest high-resolution computed tomography (HRCT).

**Results:**

CRX showed abnormal findings in 33 patients (51.5%). Most common findings were linear atelectasis (29.7%) and small pleural effusion (20.3%). HRCT showed interlobular septal thickening in four patients and crazy-paving pattern with consolidations in one patient with adult respiratory distress syndrome.

**Conclusions:**

Early CRX commonly showed linear atelectasis and small pleural effusion in Puumala virus infected patients above 30 years of age. Chest HRCT most frequently showed atelectasis and smooth interlobular septal thickening. While uncommon, early and severe pulmonary involvement can be associated with Puumala virus infection, albeit these findings are not specific.

## Introduction

The hantaviruses are enveloped RNA viruses, each carried by a specific rodent species. The most common Hantavirus in Europe is the Puumala virus, carried by the bank vole. Infection occurs by the inhalation of virus-containing aerosols from rodent excretion. People are at risk when working with hay and during crop harvesting, cleaning barns or summer cottages, cutting wood, or entering buildings infested with rodents. Puumala virus causes nephropathia epidemica, which is considered to be a mild type of hemorrhagic fever with renal syndrome [1]. Laboratory diagnosis of acute hantavirus infections is based on serology as most patients have virus-specific IgM (and usually also IgG) antibodies present in serum at the onset of symptoms [2]. The most common symptoms of nephropathia epidemica are fever, headache, backache, gastrointestinal symptoms, impaired renal function, myalgia, and blurred vision [1,3]. Puumala virus is mostly known for causing acute kidney injury. However it can affect multiple organs, including brain, heart, or lungs [4]. Respiratory manifestations, such as cough or dyspnea, have been reported in about one fifth up to two thirds of the patients infected with Puumala virus [3,5,6,7]. Severe pulmonary involvement has not generally been perceived to be a significant feature of hemorrhagic fever with renal syndrome [1,8]. When present, respiratory involvement has commonly been attributed, at least in part, to fluid overload as a result of renal failure, but there is increasing evidence that the involvement of the lung and heart is common during the acute phase of hemorrhagic fever with renal syndrome [8]. Most previous studies reported the worst imaging findings when a patient had a series of chest radiographs (CRX) done during hospitalization, and the Fleischner Society Glossary of Terms for Thoracic Imaging hadn’t been used to describe imaging findings [9]. This study aimed to evaluate the early thoracic features, at the time of admission in the emergency department, in serologically proved Puumala virus infected patients, using the Fleischner Society glossary of terms.

## Materials and Methods

### 1. Subjects

The study group was retrospectively constituted of consecutive patients who were admitted to the emergency department of a Southern Belgian hospital from 2005 to 2017 with a diagnosis of acute Puumala virus infection serologically confirmed with specific IgM antibodies. Ninety-three patients fulfilled the above criteria. Of these 93 patients, 29 patients were excluded. Twenty-one patients were excluded because they neither had CRX nor a chest high resolution computed tomography (HRCT), one because of morbid obesity and non-contributive bedside CRX, one because of synchronous trauma and six because their chest X-ray could not be retrieved from our picture archiving and communication system (PACS). Eventually, the cohort consisted of 64 patients, 45 males (range 16–77 years, mean age 45 years) and 19 females (range 18–72 years, mean age 44.6 years). Some patients had relevant past medical history: two had asthma, one had aortic insufficiency, one had cardiomyopathy, four had chronic obstructive pulmonary disease, and eight had high blood pressure.

Our institutional ethics committee gave its agreement for this study and the requirement for informed consent was waived.

### 2. Chest Imaging

Radiographs were performed using computed radiography or digital radiography. When a patient underwent several CRXs, only the first exam was recorded. Eight patients had a bedside CRX, while 56 patients had digital posteroanterior (PA) and lateral CRX. Six patients had chest HRCT, following abnormal CRX.

All HRCT had slice-thickness of 5 mm in the mediastinal and pulmonary window and thin slices in the pulmonary window (1 mm-thick slices with 8 to 10 mm gap) available for review.

### 3. Imaging Review

All imaging studies were evaluated in consensus by two radiologists (OL with 12 years’ experience and MD with 14 years’ experience in thoracic imaging). Exams were reviewed on a PACS workstation (TM-Reception-High End software, version 4.9 – Telemis, Belgium). Examination reports were not used. Earlier radiographs were used for comparison if available. HRCT images were evaluated immediately after patients’ CRX had been analyzed, and the findings were registered. Abnormalities on CRX and HRCT were reported according to the Fleischner Society glossary of terms for thoracic imaging [9]. Cardiomegaly was defined as a cardiothoracic ratio >= 0.5.

## Results

### 1. Chest Radiography

**Figure 1:**
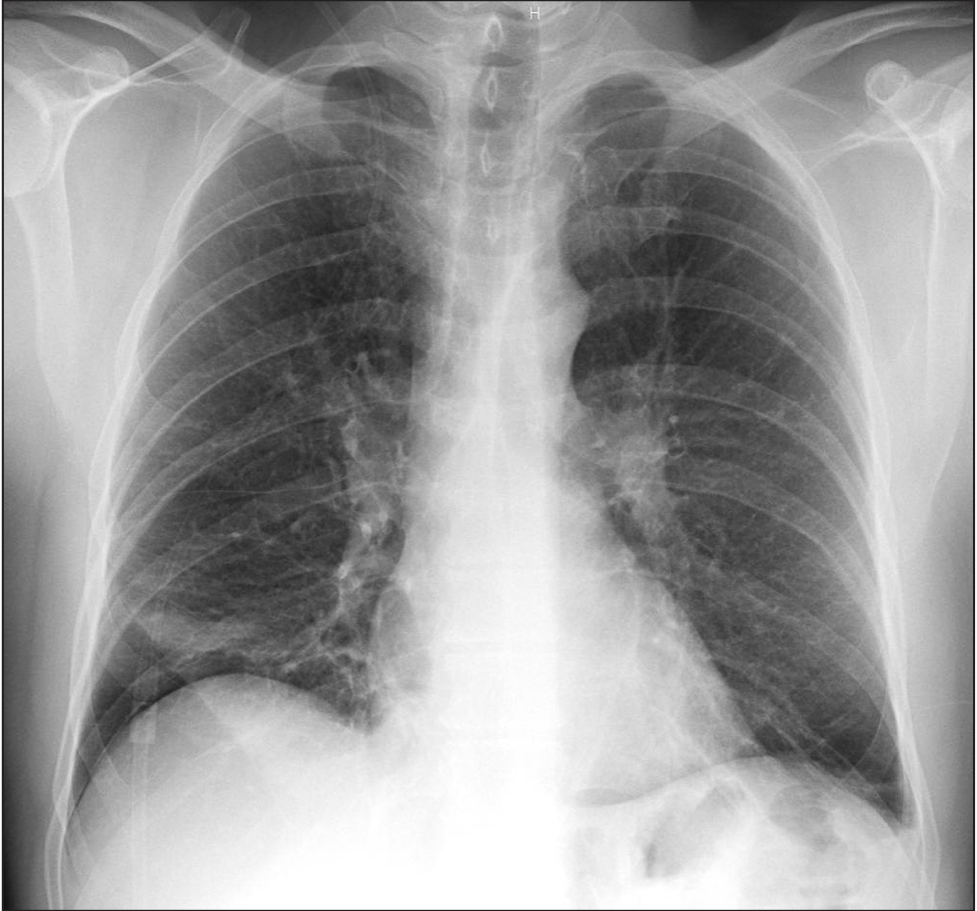
Chest X-ray (PA) showing left linear atelectasis, right subsegmental atelectasis, small left pleural effusion in a patient who presented to the emergency department with no breathing difficulty.

**Figure 2:**
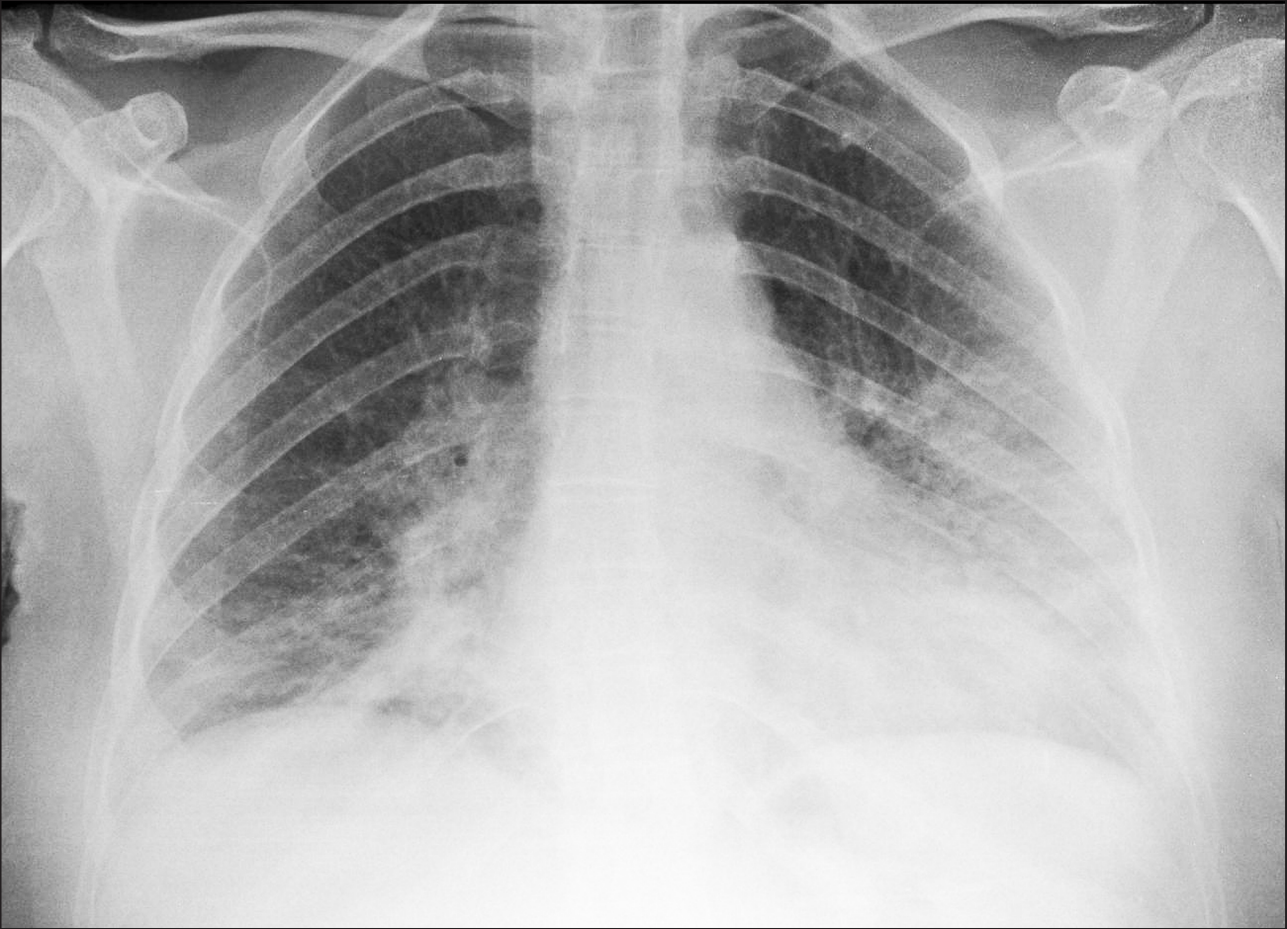
Anteroposterior bedside chest X-ray showing GGO and consolidation in a patient presenting to the emergency department with ARDS. Chest HRCT showed no pleural effusion.

Chest radiograph was performed in 64 patients (56 posteroanterior and 8 anteroposterior [AP] bedside CRX). The radiograph was performed 0–3 days (mean 0.3 days) after admission. Review of the patients’ medical records revealed that 46 patients had one CRX, seven had two CRXs, and 11 had three or more. Findings were normal in 31 patients (24 males and seven females) and abnormal in 33 patients (21 males and 12 females). The mean age of patients with normal CRX was 37.7 years while the mean age of patients with abnormal findings was 51.6 years. CRX findings are shown in Table 1, the most common were linear atelectasis (29.7%) and small pleural effusions (20.3%) (Figure 1). Atelectasis was most often limited, linear or subsegmental. Linear atelectasis was right-sided in six patients, left-sided in five and bilateral in seven patients. Segmental or subsegmental atelectasis was seen in four patients, associated with linear atelectasis in all but one case. Pleural effusions were bilateral in seven patients (11%) and left-sided only in five cases. Cardiomegaly was found in eight patients and interlobular septal thickening in five patients (Kerley lines). Ground-glass opacities (GGO) were seen in two patients. One patient presented rapidly progressive dyspnea and was admitted to the intensive care unit with ARDS (oxygen saturation was 90% using oxygen mask at a flow rate of 15L/min). His CRX showed GGO and consolidation (Figure 2).

**Table 1:** Early chest radiographic findings in Puumala virus infected patients.

Chest radiographic findings	Number of patients	Percentage of patients (%)

Atelectasis	19	29.7%
Small pleural effusion	13	20.3%
Cardiomegaly	8	12.5%
Interlobular septal thickening	5	7.8%
Ground-glass opacities	2	3.1%

### 2. HRCT

HRCT was performed in six patients (four females, two males). The time-interval between CRX and HRCT study was a median 1.8 day (range from 0 to 4 days, the four-day delay was of concern to a patient in an intensive care unit). All patients had an abnormal CRX.

**Figure 3:**
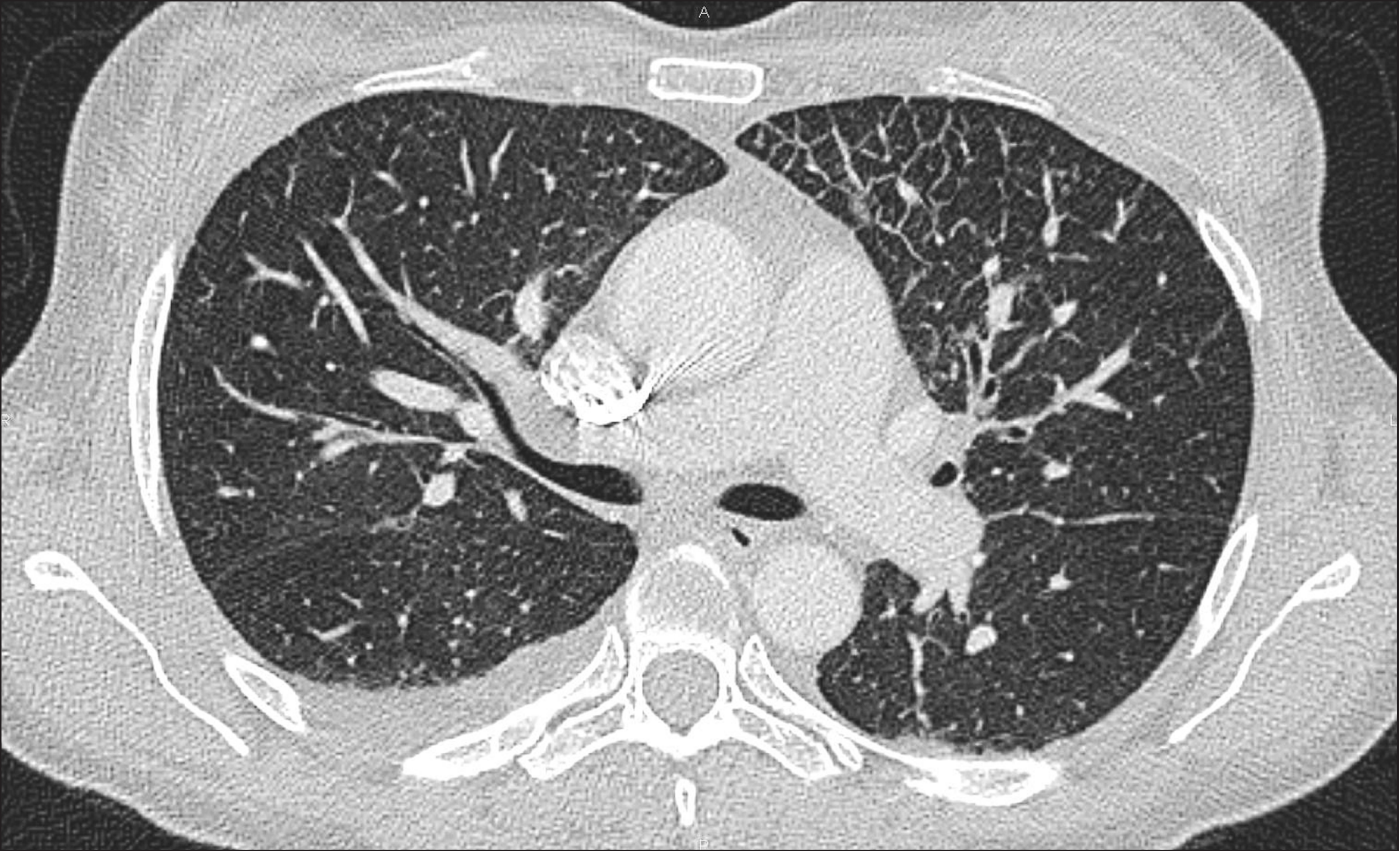
Chest HRCT showing interlobular septal thickening, with a prominent left anterior location, and small pleural effusion.

**Figure 4:**
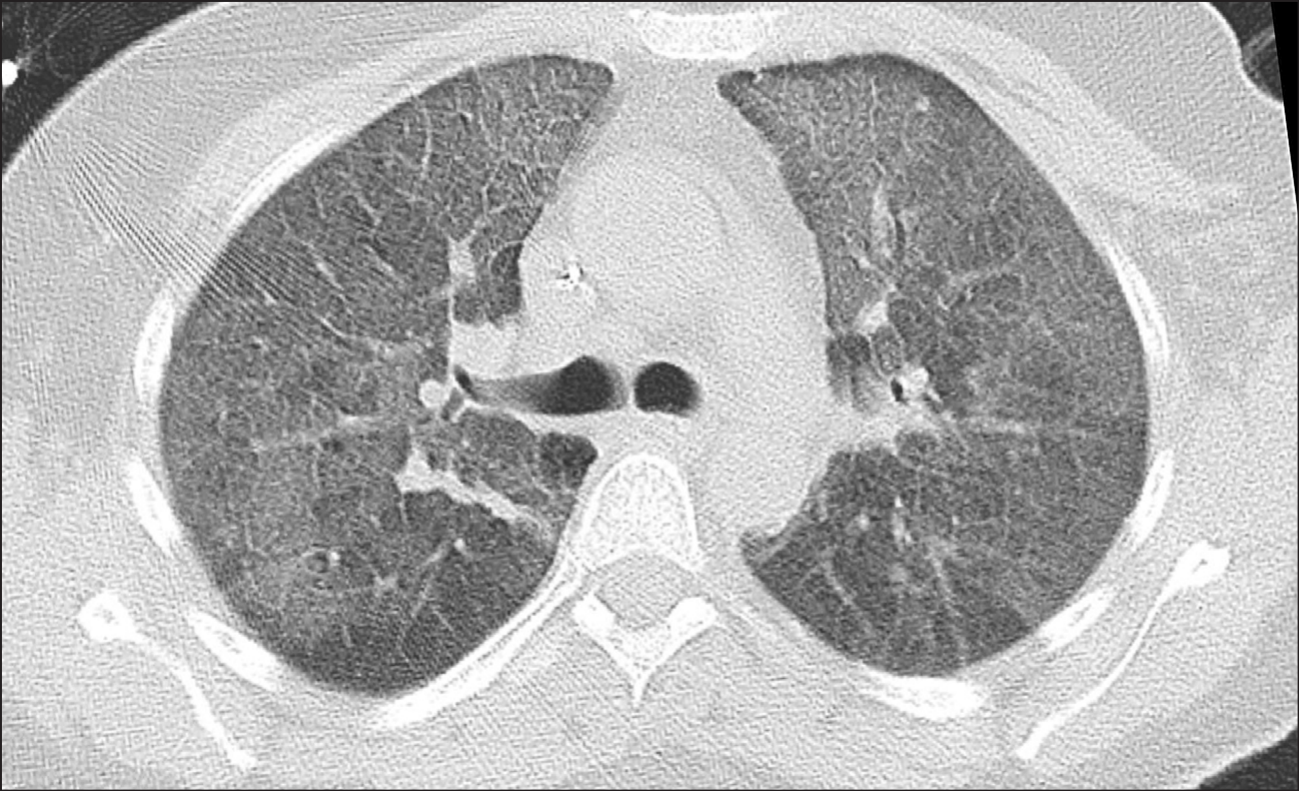
Chest HRCT showing crazy-paving pattern, and a partially imaged consolidation in the right upper lobe, with no pleural effusion in a patient with ARDS (same patient as in Figure 2).

Chest HRCT findings are shown in Table 2. Linear or subsegmental atelectasis was seen bilaterally in five patients, involving two or more lobes. Smooth interlobular septal thickening without GGO was found in four patients: two with an anterior and left-sided predominance in the middle zone of the lungs, one with a predominance in the apices, and one with septal thickening slightly more apparent in the dependent lung regions (Figure 3). Bilateral pleural effusion was observed in three patients. The crazy-paving pattern, defined as thickened interlobular septa and intralobular lines superimposed on a background of GGO [9], was found with an upper-lobe predominance in one patient presenting with ARDS, in addition to consolidations, without pleural effusion (Figure 4). An enlarged mediastinal lymph node (short axis exceeding 10 mm) was found in one patient. Bronchial wall thickening was observed in one patient and cardiothoracic ratio was increased (0.52) in one patient.

**Table 2:** Early chest HRCT findings in Puumala virus infected patients.

Chest HRCT findings	Number of patients (%)

Atelectasis	5 (83.5%)
Septal thickening without GGO	4 (66.5%)
Bilateral pleural effusion	3 (50%)
Crazy-paving pattern	1 (16.5%)
Consolidation	1 (16.5%)
Bronchial wall thickening	1 (16.5%)
Enlarged mediastinal lymph node	1 (16.5%)
Cardiomegaly	1 (16.5%)

## Discussion

While respiratory involvement is generally not perceived to be a significant feature of hemorrhagic fever with renal syndrome, CRX was performed in 77.4% of nephropathia epidemica patients who were admitted to our emergency department and found to be abnormal in 33 out of 64 (51.5%) patients with Puumala virus infection. The most common CRX findings were atelectasis (29.7%) and small pleural effusions (20.3%). Atelectasis showed no preferential side, while pleural effusions appeared to be bilateral or left-sided only. Cardiomegaly was found in eight patients and smooth interlobular septal thickening in five patients. Patients with abnormal CRX were significantly older than those with a normal CRX (mean 51.6 years versus mean 37.7 years, p < 0.0001). Moreover, all patients with an abnormal chest radiograph were over 30 years of age.

HRCT revealed linear or subsegmental atelectasis bilaterally in five patients and bilateral pleural effusion in three patients. Four patients had interlobular septal thickening without GGO, of whom two had an unusual anterior predominant distribution in the mid zone of the lungs. HRCT showed a crazy-paving pattern with a predominant distribution in the apices, without pleural effusion, in one patient with ARDS.

As previously reported, these findings are non-disease-specific [10]. Abnormal chest radiograph has previously been reported in 10–67% of nephropathia epidemica [3,5,6,10,11,12,13,14,15] and is considered common [7]. In the largest study, Paakkala et al. reported the most severe findings when multiple chest X-rays were performed and reported more pleural effusions than atelectasis [13], and so did most previous studies [2,4,10]. One study reported more atelectasis than pleural effusion in patients having HRCT examinations performed on the first day’s hospital care [10]. Focusing on early imaging, there were also more cases of atelectasis than pleural effusion in our study. We did not report any pulmonary edema. These differences are probably due to our analysis of the earliest chest imaging examination, while other studies recorded the most severe findings when a patient underwent several X-ray examinations. Indeed, respiratory involvement has commonly been attributed, at least in part, to fluid overload as a result of renal failure, and renal failure can appear or worsen within days following hospital admission [8,16]. This might partially explain why we reported more atelectasis than pleural effusion compared to studies reporting the worst findings when a patient underwent multiple chest X-rays [17]. In a previous study, the patients with radiological abnormalities were older (37 vs. 40 years, p < 0.001) than those without [11].

Only three studies reported abnormal chest CT findings [7,10,14]. In addition to pleural effusion and atelectasis or opacities, intralobular reticulations and interlobular septal thickening were the most common HRCT findings. GGO, hilar and mediastinal lymphadenopathy, bronchial wall thickening, enlarged heart size, and pericardial fluid have also been described. These lung parenchymal findings have not been reported to be disease-specific [10]. We report similar findings except for the GGO. Nevertheless, a prospective study reported no atelectasis but pulmonary edema in 21% of their patients six days on average after the onset of disease [7]. We have no explanation for this rate of pulmonary edema, except if examinations had been performed at the peak of renal insufficiency with fluid overload.

Smooth septal thickening, pleural effusions, GGO, cardiac enlargement, vascular redistribution, alveolar consolidation, enlarged mediastinal lymph nodes, and heterogeneous mediastinal fat are recognized radiological findings of congestive heart failure [18]. And hydrostatic pulmonary edema caused by intravascular volume overload has been associated with GGO, interlobular septal thickening, peribronchovascular interstitial thickening, increased vascular caliber and pleural effusion or thickening of fissures [19]. While fluid overload as a result of renal failure has commonly been suspected of causing respiratory symptoms in nephropathia epidemica patients [8], it may be tempting to do so with our patients. However, enlarged mediastinal lymph nodes were found in only one patient of our study. And the two patients presenting with smooth septal thickening in the middle thoracic zones with prominent anterior distribution, bilateral pleural effusions and inhomogeneous mediastinal fat, had normal creatinine and urea blood levels the day the CT was performed. The patient with ARDS and crazy-paving had only mild decreased glomerular filtration rate (58.5 mL/min/1.73 m²) at the time of the CT examination without signs of fluid overload, and right heart decompensation after overhydration was excluded after a normal echocardiography three days before CT. Interlobular septal thickening distribution might be of limited interest. It has been reported to have a predominantly central and gravitational distribution when caused by hydrostatic pulmonary edema [20] or to be most apparent in the dependent lung regions when caused by interstitial pulmonary edema, but this is not always the case [19]. Furthermore, septal thickening in our study showed various distributions. All in all, these findings are non-specific and may only suggest that fluid overload—while possibly contributing to some abnormal findings—is only part of the explanation.

Puumala virus is genetically closely related to hantaviruses found in America which are known to cause a frequently lethal febrile syndrome with pulmonary involvement called hantavirus pulmonary syndrome. Hantavirus pulmonary syndrome is characterized by acute respiratory distress, non-cardiogenic pulmonary edema and severe hypotension [13,21]. Puumala virus should not be confused with other hantaviruses; each strain will have a different clinical course and prognosis. Puumala virus infection is associated with mortality rates of less than 1% [2,22], while hantavirus pulmonary syndrome is associated with a mortality rate of 38% [23]. However, the demarcation between hantavirus pulmonary syndrome and hemorrhagic fever with renal syndrome is not obvious: hantavirus pulmonary syndrome cases also have renal involvement, and hemorrhagic fever with renal syndrome cases have pulmonary manifestations [1,24]. Many case reports [8,25,26,27,28] of ARDS mimicking hantavirus pulmonary syndrome in Puumala virus infected patients have been published. In some cases, mild or no renal impairment was present at the time of admission, whereas the respiratory involvement was early and severe, consistent with ARDS [8,26]. When performed, chest CT-scans showed pronounced abnormalities, including diffuse bilateral interstitial and/or alveolar opacities, dependent atelectasis or consolidation with or without moderate pleural effusions [8], noncardiogenic interstitial pulmonary edema [27], bilateral GGO [26,27] or crazy-paving pattern. The reason why some Puumala viruses have a more pronounced renal or pulmonary feature is not well understood [26].

Our study has some limitations. It was retrospective, with potential for selection bias. We focused on early chest imaging in Puumala virus infected patients and recorded the findings only of the first chest X-ray, while 18 out of 64 patients underwent two or more chest radiographs. However, patients were admitted to the emergency room at different stages of the disease, after a variable delay following the first appearance of symptoms. Some patients most probably came earlier than others, constituting a heterogeneous cohort despite our effort to focus on early imaging of the disease. Previous studies didn’t use the Fleischner society glossary of terms for thoracic imaging. It made the comparison of our results with theirs difficult, as the terminology used in former studies was not always specific [9].

## Conclusion

Initial chest imaging in patients with Puumala virus infection commonly showed non-specific linear or subsegmental atelectasis, followed by small pleural effusion, in patients older than 30 years. However, marked early pulmonary abnormalities (including crazy-paving pattern and consolidations) can be found in the case of the infrequent hantavirus pulmonary syndrome-like syndrome associated with Puumala virus infection, with mild or no renal impairment at the time of admission.

## References

[1] Vapalahti O, Mustonen J, Lundkvist Å, et al. Hantavirus infections in Europe. Lancet Infect Dis. 2003; 3: 653–661. DOI: 10.1016/S1473-3099(03)00774-6 14522264

[2] Mustonen J, Mäkelä S, Outinen T, et al. The pathogenesis of nephropathia epidemica: New knowledge and unanswered questions. Antiviral Res. 2013; 100: 589–604. DOI: 10.1016/j.antiviral.2013.10.001 24126075

[3] Settergren B, Juto P, Trollfors B, et al. Clinical characteristics of nephropathia epidemica in Sweden: Prospective study of 74 cases. Rev Infect Dis. 1989; 11: 921–927. DOI: 10.1093/clinids/11.6.921 2574903

[4] Lebecque O, Dupont M. Puumala hantavirus: An imaging review. Acta Radiol. 2020; 61(8): 1072–1079. DOI: 10.1177/0284185119889564 31805769

[5] Mustonen J, Brummer-Korvenkontio M, Hedman K, et al. Nephropathia epidemica in Finland: A retrospective study of 126 cases. Scand J Infect Dis. 1994; 26: 7–13. DOI: 10.3109/00365549409008583 7910705

[6] Nguyên AT, Penalba C, Bernadac P, et al. [Respiratory manifestations of hemorrhagic fever with renal syndrome. Retrospective study of 129 cases in Champagne-Ardenne and Lorraine]. Presse Medicale Paris Fr. 1983 2001; 30: 55–58. 11244810

[7] Rasmuson J, Lindqvist P, Sörensen K, et al. Cardiopulmonary involvement in Puumala hantavirus infection. BMC Infect Dis. 13 Epub ahead of print 12 2013 DOI: 10.1186/1471-2334-13-501 PMC4231367 24160911

[8] Rasmuson J, Andersson C, Norrman E, et al. Time to revise the paradigm of hantavirus syndromes? Hantavirus pulmonary syndrome caused by European hantavirus. Eur J Clin Microbiol Infect Dis. 2011; 30: 685–690. DOI: 10.1007/s10096-010-1141-6 21234633 PMC3075397

[9] Hansell DM, Bankier AA, MacMahon H, et al. Fleischner Society: Glossary of Terms for Thoracic Imaging. Radiology. 2008; 246: 697–722. DOI: 10.1148/radiol.2462070712 18195376

[10] Paakkala A, Järvenpää R, Mäkelä S, et al. Pulmonary high-resolution computed tomography findings in nephropathia epidemica. Eur J Radiol. 2012; 81: 1707–1711. DOI: 10.1016/j.ejrad.2011.04.049 21600717 PMC7125555

[11] Kanerva M, Paakkala A, Mustonen J, et al. Pulmonary involvement in nephropathia epidemica: Radiological findings and their clinical correlations. Clin Nephrol. 1996; 46: 369–378. 8982552

[12] Mäkelä S, Kokkonen L, Ala-Houhala I, et al. More than half of the patients with acute Puumala hantavirus infection have abnormal cardiac findings. Scand J Infect Dis. 2009; 41: 57–62. DOI: 10.1080/00365540802502629 18932105

[13] Paakkala A, Lempinen L, Paakkala T, et al. Medical imaging in nephropathia epidemica and their clinical correlations. Eur J Intern Med. 2004; 15: 284–290. DOI: 10.1016/j.ejim.2004.07.001 15450985

[14] Linderholm M, Settergren B, Tärnvik A, et al. Pulmonary involvement in nephropathia epidemica as demonstrated by computed tomography. Infection. 1992; 20: 263–266. DOI: 10.1007/BF01710791 1358824

[15] Paakkala A, Mustonen J. Radiological findings and their clinical correlations in nephropathia epidemica. Acta Radiol. 2007; 48: 345–350. DOI: 10.1080/02841850701199629 17453509

[16] Sargianou M, Watson DC, Chra P, et al. Hantavirus infections for the clinician: From case presentation to diagnosis and treatment. Crit Rev Microbiol. 2012; 38: 317–329. DOI: 10.3109/1040841X.2012.673553 22553984

[17] Claure-Del Granado R, Mehta RL. Fluid overload in the ICU: Evaluation and management. BMC Nephrol. 17 Epub ahead of print 12 2016 DOI: 10.1186/s12882-016-0323-6 PMC4970195 27484681

[18] Slanetz PJ, Truong M, Shepard JA, et al. Mediastinal lymphadenopathy and hazy mediastinal fat: New CT findings of congestive heart failure. Am J Roentgenol. 1998; 171: 1307–1309. DOI: 10.2214/ajr.171.5.9798869 9798869

[19] Storto ML, Kee ST, Golden JA, et al. Hydrostatic pulmonary edema: High-resolution CT findings. Am J Roentgenol. 1995; 165: 817–820. DOI: 10.2214/ajr.165.4.7676973 7676973

[20] Primack SL, Müller NL, Mayo JR, et al. Pulmonary parenchymal abnormalities of vascular origin: High-resolution CT findings. Radiogr Rev Publ Radiol Soc N Am Inc. 1994; 14: 739–746. DOI: 10.1148/radiographics.14.4.7938765 7938765

[21] Duchin JS, Koster FT, Peters CJ, et al. Hantavirus pulmonary syndrome: A clinical description of 17 patients with a newly recognized disease. The Hantavirus Study Group. N Engl J Med. 1994; 330: 949–955. DOI: 10.1056/NEJM199404073301401 8121458

[22] Vaheri A, Henttonen H, Voutilainen L, et al. Hantavirus infections in Europe and their impact on public health: Hantavirus infections in Europe. Rev Med Virol. 2013; 23: 35–49. DOI: 10.1002/rmv.1722 22761056

[23] Signs & Symptoms|Hantavirus|DHCPP|CDC, https://www.cdc.gov/hantavirus/hps/symptoms.html (2019, accessed 25 March 2019).

[24] Clement J, Maes P, Lagrou K, et al. A unifying hypothesis and a single name for a complex globally emerging infection: Hantavirus disease. Eur J Clin Microbiol Infect Dis. 2012; 31: 1–5. DOI: 10.1007/s10096-011-1456-y 22068273 PMC7101631

[25] Clement J, Colson P, McKenna P. Hantavirus pulmonary syndrome in New England and Europe. N Engl J Med. 1994; 331: 545–546; author reply 547–548. DOI: 10.1056/NEJM199408253310813 8041425

[26] Gizzi M, Delaere B, Weynand B, et al. Another case of “European hantavirus pulmonary syndrome” with severe lung, prior to kidney, involvement, and diagnosed by viral inclusions in lung macrophages. Eur J Clin Microbiol Infect Dis. 2013; 32: 1341–1345. DOI: 10.1007/s10096-013-1885-x 23670277 PMC7102061

[27] Fakhrai N, Mueller-Mang C, El-Rabadi K, et al. Puumala virus infection: Radiologic findings. J Thorac Imaging. 2011; 26: W51–W53. DOI: 10.1097/RTI.0b013e3181d29dfd 20736852

[28] Caramello P, Canta F, Bonino L, et al. Puumala Virus Pulmonary Syndrome in a Romanian Immigrant. J Travel Med. 2006; 9: 326–329. DOI: 10.2310/7060.2002.30014 12962589

